# The Hospital Nursing Supplement

**Published:** 1893-07-15

**Authors:** 


### "The Hospital" Nursing Mirror.

Being the Extra Nursing Supplement of "The Hospital" Newspaper.

[Contributions for this Supplement should be addressed to the Editor, The Hospital, 428, Strand, London, W.C., and should have the word "Nursing" plainly written in left-hand top corner of the envelope.]

### News from the Nursing World.

### NURSES FOR WORKHOUSES.

A meeting to promote the work of the Association for Providing Trained Nurses in Workhouse Infirmaries was held at 2, Carlton Gardens, on the 11th inst. The President, Princess Christian, was unavoidably absent, and her place was taken by Lord Wantage. Lady Wantage, Miss Louisa Twining, Mr. Bousefield, and Lord George Hamilton ably advocated this admirable association, and spoke of the need of funds for carrying on and increasing the work. After obtaining the consent of Boards of Guardians to employ trained nurses in workhouses, it is sad that their requests should constantly have to be refused on account of the inadequate supply. It is proposed to start a "Training Fund," and each subscriber of £20 will secure training for an additional nurse.

### EMERGENCIES INTELLIGENTLY MET.

Nurses in the streets or on seats alongside the route taken by the Royal procession last week had ample opportunities for viewing details of the treatment meted out to the sick or injured by the crowds around them. Fainting fits and such like were, generally speaking, disposed of excellently well, either by the police or by ordinary spectators. Water seemed invariably procurable from a military bucket hastily requisitioned, or else handed through the windows of private houses. Ice was offered sometimes, and after the patient had been served the remainder was seized by eager hands and transferred to other parched lips. Fans and smelling salts seemed to be equally plentiful, and kindly sympathy abounded. No one could fail to be struck by the improved handling of sick and wounded persons in the present day. Many untrained people are by no means untaught ones, and the way in which stretchers were used last week showed this. The humane treatment of those injured in the streets reflected credit on all concerned, and bore witness to the enormous advance which nurse training has directly and indirectly exercised in the treatment of every-day emergencies. This should obviate all danger of the confounding first-aid with the after-treatment, which is solely the affair of the qualified medical practitioner.

### INCOMPLETE.

In England we do not very often find that a lady doctor is also a trained nurse, advantageous as this would be for herself and her patients. Students can take up all the various branches of medicine straight from the schoolroom, if their parents are willing they should do so. Therefore, a girl who is still in her teens, may be quite at home in the work of the dissecting room, half-a-dozen years before a hospita would admit her as a probationer. Hence the fact that an aspirant can enter upon her medical studies whilst she is still an inexperienced girl militates against her obtaining even an elementary practical knowledge of skilled nursing. When she is qualified it is almost impossible for her to get proper instruction in the art of nursing, for it would be a terribly "unprofessional" ambition, and one which her friends would speedily put down. Yet it would assuredly help to make these young students more tender, sympathetic, and womanly, if they could acquire knowledge of humanity as well as disease. In America this has already been realised, and we find Miss Hampton uses the significant words "we see that women are beginning to look upon a thorough knowledge of nursing as an essential groundwork for their medical education." It is easy to argue that male students do not receive this experience, and this is quite true of the present race; but the middle-aged and elderly general practitioner who "walked the hospitals" before the days of training, were by no means ignorant of nursing, and many a little child and nervous woman would receive more deft attentions from their practised hands than from the fingers of the student for whom knowledge of life and of nursing are not included in the modern medical curriculum.

### PLEASURES FREELY PROVIDED.

Nurses get a great deal of pleasant recreation placed within their reach nowadays. Managers of theatres are most liberal in sending orders for their various performances, for hospital Matrons to distribute amongst the nursing staff. Tickets for the Zoological Gardens, the Albert Hall, &c., are often kindly supplied We trust that infirmary nurses are sharers in all these pleasant things. The chairman and directors of the Gardening and Forestry Exhibition have recently sent a great number of free tickets, we are told, to the metropolitan hospitals for the use of their nurses.

### THE RED CROSS FLOWER MISSION.

In times of war much sympathy is evinced towards our wounded soldiers, and all kinds of welcome gifts, such as periodicals, flowers, and fruit are sent to military hospitals for the use of the sick men who have been sent home. This enthusiasm dies out as rapidly as it rises, and military hospitals receive no offerings in peaceful times. We think, indeed, that but few of our readers are even aware that such things would be acceptable. Lieutenant-Colonel Evart, M.D., A.M.S., Woolwich, has put forth a scheme for dealing with this difficulty, and he invites systematic contributions of periodicals, papers, pictures, flowers, &c., for all the military hospitals in Great Britain, and also in India. He says that a hundred thousand admissions to hospital take place amongst the British soldiers in India every year, and those who know how eagerly news is hungered for by exiles in a foreign land will realise the acceptability of all English news. Soldiers who lie sick in English military hospitals might well be supplied with similar interests to enliven the monotony of confinement to wards. The Red Cross Flower Mission aims at supplying these and other comforts to the British soldier at home and abroad, and any one wishing to learn particulars of the proposed organisation cannot do better than write to Dr. Evart for the leaflet which he has drawn up on the subject.

### MEDALS AT GRAVESEND.

THE bronze medals worn by probationers at the Gravesend Hospital during the second and third years service are the property of the institution until the nurses have passed satisfactory examinations. After this event the medals are presented to them with their certificates, bronze being exchanged for silver medals at the end of a further period of two years, namely, when the nurses have remained for a period of five years at the hospital.

### A CAPITAL SUGGESTION.

AN excellent plan for commemorating the Royal wedding has been formulated at Yeovil. At a meeting held there last week, it was proposed by the Vicar, and heartily supported by those present, that a fund should be raised for the permanent maintenance of a district nurse. It was also suggested that the "Princess May Yeovil District People's Nursing Association" would be an appropriate title. Subscriptions were immediately promised to start the "Standing Fund." It is intended to collect £1,200 or £1,500, the interest of which would support a district nurse, any other nurses joining the association to be "self-supporting."

### DISTRICT NURSING AT STALYBRIDGE.

THE eleventh annual report of the Stalybridge Sick Nursing and Mothers' Help shows steady progress. Three nurses are employed by the Society, one of whom does night duty, and finds plenty of occupation in this important branch of nursing. One nurse has worked for nine and another for eight years at Stalybridge, and their services are greatly appreciated. A grant towards the support of a third nurse was made by the Q.V.J.I. in January, 1892, when the management of the district nursing was specially commended by one of Her Majesty's Inspectors of Nursing amongst the Sick Poor.

### ANOTHER NURSES' HOME.

THE opening of the new Nurses' Home at the Derbyshire Royal Infirmary has been satisfactorily accomplished, and the nurses are to be congratulated on the comfortable arrangements which have been made for them. During the last two years they have had many discomforts and disagreeables to encounter, and these have been met "with a heroism worthy of all praise" said Sir Henry Wilmot in a speech made at the opening ceremony. The new Home contains accommodation for forty nurses, and it cost £1,000 to furnish and £6,000 to build.

### QUEEN'S NURSES AT DARLINGTON.

THREE nurses represent the branch of the Queen Victoria Jubilee Institute at Darlington, and they are all kept fully employed. The committee has secured for them a permanent home in Greenback Road, a suitably central position. Most of the furniture and utensils needed in housekeeping have been given by tradesmen and others, who have learnt the value of the work done amongst them by the district nurses. At present the gifts received appear to have dealt chiefly with the necessaries of life, and there is still scope for those wishful to help to present a few good pictures and such pleasant things as are greatly appreciated by workers whose daily duties lie amongst ugly realities.

### WORTH CONSIDERATION.

IT is proposed by a correspondent of the *Glasgow Herald*, that a fresh plan should be tried for the treatment of those consumptive cases which it is impossible to retain in general hospitals or infirmaries. He thinks that a special hospital for this disease has obvious disadvantages, and that instead of subscriptions being raised for such a building, it would be wiser to ask for money to send the sufferers to such health resorts as may prove best suited for them, and names Davos Platz, Wiesen, and Maloja as desirable districts. If ever this scheme receives favour, it should be borne in mind that adequate attendance is as requisite as fresh air for such patients. Most of us have seen or heard of instances where men and women are sent abroad alone, to die or to recover as may be. In either case this solitude becomes an aggravation of the suffering, and although at first a person stricken may, like a wounded animal, shrink from his fellows, it is still the imperative duty of those planning to alleviate the condition of consumptive patients also to consider that in banishing them from their native land provision must be made for their being companioned suitably and cheerily, otherwise a most essential element is omitted from the scheme.

### MEDICAL MISSIONS.

AT the Medical Missionary Conference, which was held at Bombay early in the year, some valuable recommendations were made, the first being "that every medical mission should be thoroughly equipped professionally, and that to this end a hospital should be looked upon as an absolute necessity." Even more important is the following suggestion, which should certainly be adopted by every Mission Board: "That men and women sent to India as medical missionaries should invariably have a legal qualification, and that none others should be placed in charge of medical mission work."

### SHORT ITEMS.

THE diplomas awarded to the graduates at the Lady Stanley Institute for Trained Nurses at Ottawa, were presented to them on May 31st by the Countess of Derby on her last public appearance in the city.—A piece of land has been chosen for the proposed Nurses' Home at Wolverhampton, and subscriptions are solicited towards the cost of building permanent quarters for the Q.V.J.I. nurses in that town.—Miss Bertha Mason gave an excellent address at Ashton, on a recent Sunday afternoon, and described the progress which had taken place in nursing, and advocated the claims of the infirmary, and also those of the Ashton and District Sick Nursing Association in such a manner as to call forth repeated applause from her hearers.—Florence Coleman has been appointed nurse at the Langport Workhouse, at a salary of £20 per annum, with 7s. a week for rations, and 10s. per quarter in lieu of beer.—The new Convalescent Home at Withernsea was opened on July 1st, and will doubtless prove a boon to Hull and the East Riding.—Hale's "Art of Massage" has been forwarded to Miss Jelf as a prize for best answer to Examination Question for June.

### THE DISABLED NURSE.

IN acknowledging the last sum handed over to her, the Disabled Nurse adds, "the money has indeed been a great help to me; thank you for all the trouble you have taken. May I ask you again to give my sincere thanks to all who have contributed?"—We have just received 5s., with a very kind letter, from Policy 1657, for the Sick Nurse.

### Sanitation for Nurses.

By a Sanitary Inspector.

### XIII.—DISINFECTANTS (*continued*).

In addition to the two chief fluid disinfectants already mentioned, carbolic acid and corrosive sublimate, there are others which are a good deal used, chiefly on accouut of their cheapness, although their disinfecting powers are somewhat less firmly established. One of the most common of these is sulphate of iron or green vitrol. A solution of this is usually made by adding two pounds of the crystals to a gallon of water. The applications of this disinfectant are very limited. It cannot be used to disinfect linen as it produces iron mould stains, nor can it be used for washing floors as it stains wood. Its chief advantage is that in small quantities it is not poisonous if accidentally taken internally. It is, however, very bulky, and its disinfecting powers are doubtful, especially when used in its usual diluted form, though probably it is a germicide when used in a sufficiently concentrated form, and left long enough in contact with the infected matter. In some cases it forms sulphuretted hydrogen when it comes in contact with organic matter.

Chloride of zinc, called "Burnett's Fluid" because the main constituent of this disinfectant is this particular salt, used to be extensively used, but has been superseded now that our knowledge of disinfectants is so much more thorough than it used to be. It was formerly considered one of the most powerful disinfectants, but it is very poisonous. It was used for disinfecting linen and drains, but being non-volatile it is useless as a purifier of air.

Permanganate of potash, usually known as Condy's Fluid, is theoretically a good disinfectant, its action being to oxidize organic matter, but in practice it is very unsatisfactory, as it must be used in large quantities, and in a very concentrated form to produce satisfactory results. It is useful as a deodorant, and in the case of certain diseases, when it is difficult to keep the air of the sick-room sweet, some saucers of Condy's Fluid placed about the room help considerably to keep the air free from objectionable odours. It has the great advantage of being odourless itself. Strong solutions stain the hands, also linen and floors, and this fact alone would prevent its extensive use as a disinfectant.

Sanitas is a very favourite disinfectant with many people, as it has a pleasant odour, and is volatile, and does not stain at all. It is a preparation of turpentine, and is said to be a fairly good disinfectant and an excellent deodorant.

Chloride of lime owes its disinfecting powers to the chlorine which it gives off, especially when moistened with vinegar. The chlorine, however, is so extremely disagreeable to most people, and has such a irritating effect on the throat, that it cannot be widely used as a disinfectant in a sick-room, though it is valuable for disinfecting and deodorising drains and excreta; it is also the base of several disinfectant powders. Chlorine when evolved as a gas is an excellent disinfectant for empty rooms, and is preferred by some to sulphurous acid gas. For this purpose it is best obtained by placing in the room several vessels containing bleaching powder or chloride of lime, and when all openings are fastened up, such as windows, chimney, &c., then pouring some strong hydrochloric acid into the vessels immediately before leaving the room. It is very important that everything should be ready, so that the disinfector may quit the room at once, as the gas is given off directly the acid is added, and is very poisonous and suffocating. As the result of some experiments to prove the use of chlorine as a disinfectant, it has been found that (1) it acts most efficiently if "the air of the room be kept moist for some time before disinfection; (2) disinfection in dry air is uncertain; (3) no less percentage than 5·38 chlorine per 1,000 of air in room to be disinfected can be considered efficacious; (4) chlorine fumigation carried out under the best conditions may fail to disinfect spore-holding material covered over, or lurking in chinks and cracks; (5) in all cases chlorine is far more efficacious than sulphurous acid gas." As regard the quantity of chlorine gas necessary, the following proportions are recommended, viz., to every pound of chloride of lime, one and a half to two pints of hydrochloric acid should be added, and these quantities will be sufficient for every 1,000 cubic feet of space. Ordinary pottery basins should be used, not metallic vessels, as the acid will act on these last, and it is recommended that the basins be placed at various elevations about the room so as to ensure the gas being diffused throughout the whole space. In using chlorine gas as a disinfectant, it must be remembered that it also possesses very powerful bleaching properties, and hence coloured articles should not be left in the room, but care must be taken to thoroughly disinfect these by some other equally effectual measures. Under some circumstances, especially if the room be large, it may be a little difficult or troublesome to render the air sufficiently moist for the chlorine gas to have its maximum effect. Some authorities recommend that "when a room is to be disinfected, a little time before the actual disinfection a tub of boiling water should be placed in it, so that the steam may wet the walls and the air." This sounds very well in theory, but we doubt very much whether in practice a tub of boiling water would moisten the air more than very slightly indeed, much less the walls. Hence, if chlorine gas is adopted, and it is desired to make the disinfection of the room as thorough as possible by previously rendering the air moist, we think some other means than the tub method must be adopted, and that the direct generation of steam from kettles filled half full of water and kept boiling in the room for some hours previous to the disinfection by chlorine gas will be necessary. There is no doubt that the fact that moisture is required for chlorine gas to have its maximum effect detracts from its usefulness as a disinfectant for rooms after infectious disease. For this reason alone it is less useful for the general public, who require a disinfectant which is at once thoroughly effective and simple of application such as sulphurous acid gas, which of late years has been prepared ready for use in a very simple form, which can be readily applied by anyone. Sulphurous acid gas is most commonly produced by burning sulphur in an iron vessel placed over water to prevent any risk of burning the floor. To insure the complete combustion of the sulphur, some methylated spirit is put in the vessel, or sometimes simply shavings, or shavings moistened with methylated spirit are used for the same purpose. The usual quantity of sulphur recommended is one pound for every 1,000 cubic feet of space, which produces 11·7 cubic feet of sulphurous acid gas, and many authorities on the subject do not consider this sufficient to destroy germs of infectious disease, and hence it is considered by some as a doubtful disinfectant, since, under the most favourable circumstances, the air in the room does not contain more than 10 per cent. of sulphurous acid gas.

Sulphurous acid gas has been liquified, and has lately been so prepared by Messrs. Brake, Roberts, and Co., of Stratford, E., in tins, each containing 20 ounces of sulphurous acid gas, stated as equal to double that weight of sulphur. When the room has been prepared in the usual manner, a piece of lead is removed from the tin and the fluid resumes a gaseous form and fills the room. Another form in which sulphur is prepared for fumigating rooms is in the shape of candles, and known as "Seabury's Sulphur Candles." These are composed of a cake of sulphur containing two or three wicks and saturated with nitre. These wicks are readily lighted, and the sulphur is vapourised gradually.

### Educational Standards for Nurses.

By Miss Isabel A. Hampton, Superintendent of the Johns Hopkins Hospital, Baltimore, Maryland.

### I.—HOSPITAL, DISTRICT, AND PRIVATE NURSING.

While fully appreciating the honour done me it has not been without much hesitancy that I have undertaken to express my views upon so important and complex a subject as "The Standards of Education for Nurses." The subject is important because it deals with the problems of health and disease, life and death. It is complex because so many complicating factors and interests must be taken into consideration. The social problems of human misery and suffering, and how best to alleviate them, have been wonderfully worked out since the days when Charles Dickens first began to exert the power of his genius upon the mind of the public, in order to bring it to an active sense of its responsibility in such matters, and perhaps in no branch of philanthropy has the change been so marked as in the care of the sick of all classes in all countries. And when we consider the few years which have elapsed since the modern system of nursing has been introduced, and contrast the present conditions with those which formerly prevailed, we might at first sight perhaps be excused if we regarded our present methods with some complacency instead of all the time struggling to find room or ground for improvement. But with progress going on in every branch around us, are we alone to stand still? The present history of hospitals in America shows that the hospital nursing is, with a few exceptions, already being done by the members of regularly organised schools for nurses, and where such schools do not exist steps are being taken for establishing them. We find that the demand for trained nurses is steadily on the increase for cases of sickness in private families, and what is still more important, that district nursing is being introduced in almost every large city in the country. Then, too, missionary boards are requiring that their women for foreign work shall prepare themselves by receiving a course of training in nursing. Lastly, when we see that women are beginning to look upon a thorough knowledge of nursing as an essential groundwork for their medical education, we cannot but be convinced that training schools for nurses, and trained nurses are established facts, important factors in hospitals, houses and the community at large.

### Quality of Work and Workers.

In considering the standard of education requisite for such workers we have to consider (1) the kind and quality of the work required, and (2) the order of woman necessary to meet such requirements. In the daily routine of a hospital with its variety of patients, the work of a nurse, even while herself receiving instruction, is not without its immediate results. The hospital is her workshop in which she must first serve an apprenticeship and from the day she enters it, the preservation of human life and the alleviation of human suffering are to some extent delivered into her hands. Can a woman, in any other kind of work which she may choose for herself, find a higher ideal or a graver responsibility? Where human life and health are concerned what shall we term "the little things?" Let us not therefore, whether we be teachers or pupils, think lightly of any of the details of our work, and let the young nurse remember in her new and arduous duties that from the very beginning she is accomplishing something, and that unless her part is thoroughly done the whole work will be incomplete. Again in the progress that medical science is making, she has her allotted part to perform. To be sure, she is only the handmaid of that great and beautiful science in whose temple she may only serve in minor parts, but none the less is it her duty to endeavour to grasp the import of its teachings that she may fulfil wisely her share. to requires, for instance, more than mechanical skill on the part of a nurse to understand the preparations for an antiseptic operation, full of significance as it is in every detail, and the saying that "dust is danger" must have a bacteriologically practical application in her mind. Nor can just any one appreciate the full meaning of the physician when he says, "the nursing will be half the battle in this case." For the simple performance of nursing work such knowledge is requisite, but when the wider duties of either head nurse in a hospital, or principal of a school for nurses are assumed, where one must not only know but be capable of imparting that knowledge to others, then the responsibilities become proportionately greater.

### Private and District Nursing.

Turning from hospitals to consider the requirements for this work elsewhere, we find that nursing in private families, and district nursing among the poor in their homes, are the two great fields in which nurses will be principally occupied. Here the nurse is frequently even more closely identified as the physician's lieutenant, for whereas in a hospital a doctor is usually within ready call to render either advice or assistance, on the other hand, in private practice, her knowledge and skill in the absence of the physician must be depended upon in critical illnesses, or unlooked for complications, until his aid may be secured. To this part of the work in particular may be applied the following words taken from a physician's address dealing with the relation of the nurse's work to that of the physician, "The hands of a nurse are the physician's hands, lengthened out to minister to the sick. Her watchful presence at the bedside is a trained vigilance, supplementing and perfecting his watchful care; her knowledge of his patient's condition an essential element in the diagnosis of disease; her management of the patient, the practical side of medical science. If she fails to appreciate her duties, the physician fails in the same degree to bring aid to his patient." In district nursing we are confronted with conditions which require the highest order of work, but the actual nursing of the patient is the least part of what her work and influence should be among the class which the nurse will meet with. To this branch of nursing no more appropriate name can be given than "instructive nursing," for instructive in the best sense of the word it should be. Such, briefly and imperfectly outlined, are some of the chief factors to be considered in educating women to care for the sick.

### Special Qualification.

Realising then the kind and quality of the work to be done, we pass on to the consideration of the order of woman required to perform such duties, and those of us who have had much experience with nurses and know all we would have them to be, and how much they really must be, as the various classes of women pass in review before our mental vision, will be inclined to agree with the writer of a letter which came to me a short time since. After asking me to recommend a head nurse for a hospital, and enumerating at length the qualities she must possess to be successful, he concluded with the words, "in short we require an intelligent saint." The idea still prevails in many minds that almost any kind of woman will do to nurse the sick, and that the woman who has made a failure of life in every other particular may as a last resource undertake this work. After many years of continuous work among patients and nurses I am convinced that a woman to become a trained nurse should have exceptional qualifications. She must be strong mentally, morally, and physically, and to do thorough work she must have infinite tact, which is another name for common-sense.

She should be, as one of the women of Queen's Gardens in Ruskin's "Sesame and Lilies," or such an one as Olive Schreiner defines her, when she says, "A woman who does a woman's work needs a many-sided, multiform culture; the heights and depths of human life must not be beyond her vision; she must have knowledge of men and things in many states, a wide catholicity of sympathy, the strength that springs from knowledge and the magnanimity that springs from strength." Only in so far as the women of our training schools attain to this standard will the institutions and communities in which they labour, feel and show forth the influence of that "sweet ordering, arrangement, and decision," that are her chief prerogatives. What class of women have the same practical privileges of learning the means to be used for the prevention of disease or of realising their importance? Who is so competent, or who has greater opportunities for daily practising than herself, and teaching them to others? and intelligent she ought and must be to do this wisely; otherwise she is a mere machine, performing mechanically the task before her, not knowing or caring why or what it all means, and the public loses thereby the services of one who should be valuable in showing them something at least of the beauty of the laws of hygiene, and their application, and who can fortify her teaching with scientific facts.

*(To be continued.)*

### Associations for the Supply of Cottage Nurses.

A DRAWING-ROOM meeting was held last week to consider the establishment of new associations for the supply of cottage nurses on the Holt-Ockley system. Lord Brassey presided, and a good many ladies were present. Explaining the objects of these associations, Lord Brassey said they were intended to bring good and skilled nursing into country districts, and were originally instituted by Miss Broadwood in 1883 at Ockley, in Surrey, and were now spreading all over England. The system was efficient and economical, and the good already done incalculable.

Miss Broadwood described the work of the Ockley Association, and said that while all the nurses employed had received some training, skilled hospital nurses were not engaged, first because of the high fees, and secondly because they would not undertake the rough cottage work in addition to the nursing. It was also found that as a rule a highly skilled nurse was not needed for the majority of cases of illness in the villages. The scale of yearly subscriptions was as follows: 2s. for labourers, 3s. for artizans, gentlemen's servants, &c.; 5s. for farmers and tradespeople, and 10s. for gentry. At the time when the nurses' services were required, weekly fees for the same amounts were charged. Subscribers could claim a nurse as a right, while non-subscribers could only take their chance. Strong and healthy women were employed, able and willing to face all weathers, and to stand rough work. The Holt-Ockley scheme was not a charity; it was intended to be more of the nature of a huge benefit club, binding all classes together in a common bond of mutual kindness and help. The salaries were not so high as those commanded by district nurses in towns, but greater freedom of life and intervals of rest were appreciated by the nurses as compensating advantages.

While acknowledging the fact that the qualifications which go to make a good district nurse are of a different character from those which are desirable in a nurse whose work lies amongst patients of a higher order in the social scale, we should like to point out that the responsibilities which fall to the share of the former are often of a very serious nature, and that in country districts where medical aid is far to seek the damage which may be done by half-trained nurses is incalculable. We should like to know what amount of training is considered essential for those nurses who are employed by the Holt-Ockley Associations.

### Select Committee on Midwives.

THE Select Committee met for hearing witnesses on Friday, June 30th, and examined Mr. Braxton Hicks (coroner), Dr. Bott, of Brentford, and Dr. Emmerson, of Biggleswade.

Mr. Braxton Hicks considered that reform was called for, that no women should be allowed to act as midwives unless they were properly qualified for their duties, and that no midwife should be allowed to give a certificate of any description. He mentioned his suggestion that coroners should be in the legal, not in the medical profession, but should have a medical assessor to inspect and make post-mortem examinations if necessary in all cases where a medical man had not been able to give a certificate. He believed that there was a certain amount of criminal abortion-procuring carried on through the agency of midwives.

Dr. Bott, of Brentford, remarked that as Medical Officer of Health he was able to say a good deal about the condition of things in his own town. About ten years ago two certified midwives were engaged by a charity to attend the poorer classes on a partly provident system, and that they had taken up nearly all the work of a number of untrained women who had previously practised there, and that they now attended about half of all the confinements in the town. A small number of untrained women still practised there, about whose conduct he had grave suspicions. Owing to poverty, to the daily absence of the mothers, who worked at making punnets, the children were much neglected, and the infantile mortality very heavy.

He was always called in to the trained midwives' cases, and practically found that although a natural labour could not be defined for their instruction, they always sent in time, and he thought that the better attended the women were during labour the less would be the mortality amongst the children. He thought they should be registered similarly to medical men after proper training.

Dr. Emmerson, of Biggleswade, had seen some very bad cases of malpraxis. The population—a purely agricultural one—was very poor, and only paid the midwives 2s. 6d. to 4s. No midwife in the district was trained, and there were six or seven of them. Their attendance was purely a question of money. There was considerable difficulty in obtaining parish orders, and practically no order was ever given for a first confinement, nor for others unless a bad time was to be apprehended. He was very seldom sent for by a midwife, and he hoped these poor women would not be deprived of their means of living. He thought it desirable they should be trained and registered.

### Appointments.

[It is requested that successful candidates will send a copy of their applications and testimonials, with date of election, to THE EDITOR, The Lodge, Porchester Square, W.]

CUMBERLAND INFIRMARY.—Miss Linda Ditcham has been appointed Matron of this Infirmary. Miss Ditcham was a lady pupil at Guy's Hospital, and for the last four years has had charge of a ward at Cumberland Infirmary. We congratulate her on her promotion to her present responsible post.

OAKELEY'S HOSPITAL, BLAENAU FESTINIOG.—Miss Annie Abbit has been appointed Matron of Oakeley's Hospital. She has had considerable experience in private nursing, and for the last two years has been Head Nurse at the Carnarvonshire and Anglesea Infirmary, Bangor. We wish her a successful career in her present post.

### Rabies and Glanders.

### I.—RABIES.

On Wednesday, July 5th, Dr. Charles S. Sherrington, Professor Superintendent of the Brown Institute, gave the first of a course of four free lectures in the theatre of London University, Burlington Gardens, at five o'clock. Although the above two subjects were announced, the Professor found that time only allowed of the treatment of one—Rabies.

Dr. Sherrington stated that rabies presented itself under three aspects. (1) As it occurs amongst dogs sent to him for relief in the hospital; (2) post-mortem examination when death is believed to be due to rabies; and (3) experimental rabies.

It was observed that, whereas we distinguish the same disease according as it occurs in man or animal as hydrophobia and rabies, abroad the latter term is alone used for all cases.

Rabies is especially a disease of dogs, and rarely attacks other animals. However, oxen, sheep, swine, deer, and rabbits are liable to it, and within recent years there have been two epidemics amongst deer—in 1887 amongst those in Richmond, and in 1888 at Ilkworth Park in Suffolk. It is curious that, although rabbits and guinea-pigs are so closely allied in many ways, the former are liable to rabies, whilst no case of it has been known in the guinea-pig. As regards cats, they are very rarely affected in this country, whilst in Belgium and Russia rabies is common amongst them, and they rank next to the dog in frequency of attack.

The cause of the disease is still very obscure, and dogs who have been kept carefully shut up in a garden alone have been known to contract it. The lecturer said that though the clinical aspects are multiform, rabies can be broadly divided into two types: (1) Dumb rabies, (2) violent rabies.

In Dumb rabies, the clinical symptoms in well-marked cases are easily recognised, whilst in others they are less clearly established. When a dog is admitted for dumb rabies, it shows no signs whatever at first, and the characteristic symptoms appear gradually afterwards. These are as follows: Although the animal is quiet it is distinctly uneasy and restless, slightly salivated; on opening the mouth it is seen to be festooned with thick viscid saliva. There is a hang or droop of the lower jaw, the eyes are restless, the pupils very much dilated, the hair is harsh to the feel, and the dog appears to be in pain. The dog is generally noticed to be ailing three or four days before his owner brings him to the hospital, and during that time eats nothing though he drinks plenty of water. A dog in this condition very rarely barks and is not specially morose when interfered with. The disease generally ends in rapid sinking and death.

In Violent rabies one of the earliest symptoms is extreme combativeness. One dog in particular that had come under the Professor's notice was confined because he fought every dog he met—but he managed to escape and made his way to a village where a fair was going on, again fighting every dog he met and killing some poultry. In fact, combativeness is the chief symptom of violent rabies. The dog refuses all food, though he drinks water freely. After about forty-eight hours he becomes very morose, snarling from time to time, though he lies quietly. Next salivation appears, the dog refuses both food and water, there is great weakness in the hind legs, the lower jaw droops, and the animal invariably dies.

Dr. Sherrington next described in detail the symptoms produced in animals inoculated with rabitic virus, and from these observations, together with the clinical symptoms observed in animals attacked by rabies, gave the following as the chief signs by which the disease could be diagnosed:—

1st. The bark is high pitched and peculiar.

2nd. Refusal of food, but not of water.

3rd. Salivation, which is usually quite an early sign, though not peculiar to rabies.

4th. There is no recovery, and the animal always dies. There are, however, three cases of supposed recovery, but the evidence of the disease being rabies is not very conclusive.

It is very difficult to recognise rabies by post-mortem appearances, because though there are certain well-established signs, these are by no means always present. The condition of the stomach is a valuable sign, it shows frequently slight hæmorrhages and inflammation, with erosions in the mucous membrane, which are probably due to the harm done by the various things a rabid dog will eat, such as stones, sticks, straw, &c., so that the stomach has been found actually packed full of these things. As this is done early in the disease, it quite accounts for the state of the stomach. The mucous membrane of the larynx is extremely congested, but this does not cause the peculiar high-pitched bark, as the mucous membrane is congested also in dumb rabies, and the alteration in the bark has been found to be due to nervous changes.

Dr. Sherrington spoke with approval of the "muzzling order," and said it had certainly stamped out rabies in certain districts.

In conclusion, the lecturer showed a map of England in which the districts where rabies is most prevalent were coloured blue; these were within the last fifteen years, around London, in South Lancashire, in Cheshire, South-West Yorkshire, Staffordshire, Derbyshire, and Nottingham.

### Everybody's Opinion.

### LECTURES.

"A Puzzled Probationer" writes: May I call your attention to the kind of lectures we nurses are condemned to listen to and expected to understand? I have recently entered as a probationer in a certain London hospital. I have had a liberal education and trust I am not more stupid than the average nurse. Being warned as to the advanced nature of the lectures I should have to attend in the course of my first year's training, I studied elementary books on anatomy and physiology beforehand. I went to the first lecture full of hope and desirous to learn, but I left it addle-brained and broken-hearted. All I can remember about it are the words "Zona pellucida," "vitelline membrane," "discus proligerus," "germinal vesicle," "Graafian follicle," "blastoderm," "epiblast," "hypoblast," and various other "blasts," "Chorion," "Amnion," "Allantois," and other names like those of the gods of the heathen. Surely to open a course of elementary lectures by advanced embriology is an Irishman's way of beginning at the beginning.

### STAFFORDSHIRE INSTITUTION FOR NURSES.

Miss Mary Shirley, the Lady Superintendent, writes from Stoke-upon-Trent: May I ask you to correct a very curious mistake in last week's Hospital? In your report of our institution, you say our nurses have lived in lodgings between cases. Such is not so. We have a very lovely home, with accommodation for sixteen nurses. What we are going to build is a cottage, where we can isolate those nurses who cannot be kept in the home during sickness of any infectious kind.

### Death in our Ranks.

Miss Edith Hawksworth died on July 5th of typhoid, contracted whilst working, by her own special desire, amongst the sick poor at Worthing. She held the post of Sister-in-Charge of the Sussex County Hospital Nursing Home, and had been connected with that hospital since 1886. Miss Hawksworth was greatly beloved and is universally regretted.

Miss M. E. Atkinson died of pneumonia on July 7th at Manchester Southern Hospital, where she was Sister of the women's ward. Her death is deeply regretted by a large number of friends.

### A Novel Competition for Nurses.

IN the course of the discussion on Nurses' Homes which took place at the International Congress at Chicago, Dr. Billings, the President, expressed the view that it was about time that women manifested the latent power claimed for them in a practical and definite form. He pointed out, for example, that it had been left to man—an impotent weak creature as women knew him to be—to design and erect homes for nurses and training school buildings. Architects and male experts had exhausted their ingenuity in designing such buildings, and he thought that Congress was a very appropriate platform from which to invite the exhibition of woman's latent power by urging the superintendents to elaborate plans and enumerate each item which a model nurses' home and training school should contain. We have had much pleasure in falling in with Dr. Billings' suggestion, and have decided to organise a competition which will be open to any woman engaged in nursing from any part of the civilized world. The following details of the proposed competition will no doubt be of interest.

ATTACHED TO A HOSPITAL OR UNDER SEPARATE MANAGEMENT.

Two prizes, namely, a first prize of $75 (£15,) for the best plan sent in for competition, and a second prize of $25 (£5) are offered by the Editor of THE HOSPITAL, London, England, for competition amongst lady superintendents, matrons and nurses in the British Empire, the United States, and the countries of Europe.

Competitors must send in the proposals in type-written form, which must consist of two parts: (1) A statement setting forth categorically all the rooms and accommodation which are necessary in order to make the proposed nurses' home a model of what such an establishment should be where room is provided for from fifty to one hundred nurses; (2) A second statement and plan prepared in similar form which provides accommodation for from one hundred and fifty to two hundred nurses. Each of these specifications (1) and (2) must be accompanied by plans drawn to a uniform scale, and must explain how the proposed home for nurses can be extended, should the growth of the establishment render such a step necessary

*Conditions.*

1. The competitors must send their names and addresses by November 1st, 1893, to Dr. J. S. Billings, U.S. Army, Washington, D.C., and all plans and proposals presented for competition must reach Dr. Billings not later than *January* 1st, 1894.

2. Dr. Billings, Dr. Henry M. Hurd, Superintendent of the Johns Hopkins Hospital, Baltimore, Md., and Miss Sophia S. Palmer, Superintendent of the Garfield Hospital, Washington, D.C., are appointed as the judges of the papers and plans submitted in this competition, and their decision will be final. Should the papers and plans submitted demand such a course, the judges reserve the right of withholding any award.

3. All proposals must be type-written on one side of the paper only, and all plans must be drawn to a uniform scale of one-eighth inch to one foot.

SPECIAL NOTE.—It is proposed to publish an account of the competition, with a selection from the competing plans, such publication to be undertaken by the Scientific Press (Limited), 428, Strand, London, W.C., at their option, to whom papers and plans will be sent by the judges after they have made their award.

### Notes and Queries.

**Queries.**

(157) *Rome.*—What is the name of the English Nursing Sisterhood at Rome ?—*Wanderer.*

(158) *Women's Work.*—Are women dispensers ever advertised for ?—*Dispensing.*

(159) *Q.V.T.I.*—Are maternity nurses received as Queen's nurses ?—*A Monthly Nurse.*

**Answers.**

(157) *Rome (Wanderer).*—The first English nuns to take up their quarters in Rome were "Mary's Company," or "The Little Company of Mary," who settled there eleven years ago.

(158) *Women's Work (Dispensing).*—Certainly they are. A lady dispenser is required now for the Women's Hospital, Chelsea.

(159) *Q.V.T.I. (A Monthly Nurse).*—The rules for Queen's nurses can be obtained from the Superintendent, St. Katharine's Hospital, Regent's Park.

### For Reading to the Sick.

MARRIAGE.

ALL England has been occupied of late in rejoicing at the Royal marriage, and even the sad and suffering may well turn their thoughts in the same direction, and try to lighten their sorrows and forget self in sympathising with the young pair to whose earthly course they wish God-speed.

O Lord of life and love,
Come Thou again to-day,
And bring a blessing from above
That ne'er shall pass away.

O bless, as erst of old,
The Bridegroom and the Bride,
Bless with the holy stream that flowed
Forth from Thy pierced side.

Many and noble are the ancestors from whom both the Duke and Princess are descended. They are the son and daughter of a long line of kings, and may themselves (please God) wear the Crown of England in due course. But a far nobler portion is theirs; they are the children of the King of kings, and by their birthright in baptism are heirs to the Kingdom of Heaven.

And in this "high calling" we too have our part, we are all God's children by adoption and grace. He has promised to all—rich or poor, young or old, gentle or simple—a golden crown, with untold riches and treasures, in the "many mansions" prepared for those who love and faithfully follow the "Prince of Peace" through the trials of this troublesome world. "Eye hath not seen, nor ear heard, neither hath it entered into the heart of man to conceive" what is laid up for us by our Father, the King of kings. It is always pleasant to think of the wedding of a well-assorted couple. The flowers, the lights, and the beautiful garments of the guests make a charming spectacle and point to the "marriage feast of Holy Scripture, where the bride is arrayed in fine linen, clean and white; for the fine linen is the righteousness of saints." But we have warnings in the two other weddings spoken of in the Gospels. The man who had not on the "wedding garments" was cast out, and the foolish virgins who were sleepy and idle and had let their lamps go out, were left to lament "Too late! too late!" as they saw their companions pass in to the marriage. We must clothe ourselves with humility and meekness, faith, and love, the "marriage garments," and with purity and cleanliness, "the righteousness of saints." We must watch, for we know not in what hour the Lord cometh, and so with our lamps burning we shall be ready at any moment to rise up and follow the Lamb withersoever He goeth. The angel said to St. John in his revelation, "Write, blessed are they which are called to the marriage supper of the Lamb."

### London Hospital.

THE result of the annual examination for probationers, which took place at the London Hospital Medical College on the evenings of June 29th and 30th and July 1st, was as follows:—First prize, Probationer Irene Webb; second prizes, Probationers Ann Finnemore and Eliza Smith. Honorary certificates were gained by Probationers Louisa Lessey, Elizabeth Venning, Maria Walton, and Ellen Patterson. Number of probationers competing, 76.

OXFORDSHIRE COUNTY COUNCIL.

THE County Council of Oxfordshire have offered scholarships to train nurses recommended by local nursing associations at approved training institutions. The scholarships are of the value of £10.

### The International Nursing Congress, Chicago.

(By Our Special Commissioner.)

### II.—DISCUSSIONS ON NURSING TOPICS.

Last week we gave an outline of the proceedings at the Congress, and we now propose to summarise the principal discussions in the Nursing section. This section was very largely attended, and certainly constituted probably the largest gathering of nurses representative of more countries and centres of training than have ever assembled before in the history of the world. Miss Hampton grouped the papers admirably, and kept the proceedings well in hand. A strong chairman who talks little may possibly diminish the number of speakers, but secures that all the business is transacted within the allotted time. Hence, the Nursing section was in this respect in marked contrast to that of some other sections, where the proceedings were frequently unduly prolonged. The proceedings opened with Miss Lumsden's paper on "Nursing in Scotland," which was followed by one on "The Proper Organisation of Training Schools in America," by Miss Darche, of the New York City School for Nurses. In the discussion which followed the chief points raised referred to the importance of personal service, and the working of training school registries. Miss Mary Levins insisted that no national association or local alumni could be successful, unless each nurse and member felt a personal pride and pleasure in furthering the interests of her own organisation and of her fellow workers as a class. There was a privilege in personal service which caused each one to try to attend the meetings, and do all they could to further the interests of the association as opposed to those of individuals, which all must recognise. Mrs. Nickson declared that in Chicago it was a common saying that only paupers and millionaires could enjoy the luxury of a nurse. To remedy this evil the Greer Fund had been instituted in connection with the Illinois Training School, which was modelled on the system of the Dubois Fund in New York, with the object of sending out nurses for smaller payments to meet the case of families whose means were moderate. The President of the Illinois Training School had suggested that each graduate should agree to take one case a year under the Dubois and Greer system for a payment of from twelve to forty shillings (three to ten dollars) per week, according to the means of the patient. In order to prevent this system from being a tax upon any nurse's good nature, arrangements are made that each nurse shall undertake a case for a definite time, say a week, when her place is taken by another nurse, and so on, until the patient no longer needs attention. It appears that many nurses give their services at a very low figure in a private way, but it was the general feeling that some organisation like the Dubois and Greer Funds was needed throughout the large cities of the United States, not only in the interests of the public, but as a protection to nurses as a class.

### Training School Registries.

Miss McKechvine, of Kentucky, introduced the subject of a school registry managed by the superintendent of a training school, where there are set rules and set charges. Miss Darche explained that these registries were generally confined to nurses required for obstetric and contagious diseases. Most schools have followed the original plan of the Belle Vue, where the standard ranges from £4 to £5 (20 to 25 dols.) per week. In the smaller towns, where living is cheaper, the rate is usually £3 or 15 dols. per week. Some nurses urged that the training school or hospital had no right to decide what a nurse should charge, as each nurse ought to have the privilege of setting her own price, seeing that hers is as much professional work as that of the physician. Miss Darche, however, pointed out that it was quite optional whether a graduated nurse belonged to a registry or not. If she belonged to it, she must conform to the rules. If she preferred to be independent, and to obtain her cases independently of the registry, then she could charge what she pleased. Another nurse stated that the graduates of the training school found it a great protection to have the rates fixed. A nurse's charges were often contested, but if she can produce the scale issued by the training school registry, the patients accept this document as final, and pay the fee without further trouble. Miss Darche further explained, in reply to Miss Shield of Indiana, that, although the nurses were not permitted to charge less than the directory fees, they were at liberty to give a whole week, or a few days, at their discretion, for nothing. Miss Rogers urged that the older nurses were entitled to higher fees for private work than those with less experience, and it was admitted that, whereas a nurse could not charge less than the scale, she was at liberty to charge more by arranging with the registry not to take a case for less than the maximum charge of £6, or 30 dollars per week. Miss Darche further explained that the older nurses find, after awhile that "they do not need the aid of the registry at all, as cases come to them in sufficient numbers independently, and then they charge their patients what they consider fair." Miss Darche, therefore, thought the registries were a great protection to the younger nurses, and that the majority of nurses like to be associated together through their agency. Miss Lett, of Chicago, stated that in all schools the superintendents allow the nurses from a week to a fortnight to do as they like in. This year she had had two weeks given to her to devote to people that could not afford to pay. She was of opinion that this spirit ought to be encouraged in every training school. (Applause.)

### Matrons and Lady Superintendents.

A paper entitled "Nurses as Heads of Hospitals," by Miss E. P. Davis, Superintendent of the University of Pennsylvania Hospital, created an interesting discussion. Miss Rogers, of Washington, insisted that no lady ought to undertake the superintendency of a hospital without special training. In the smaller hospitals the lady superintendent had not only to supervise the nursing, but also the housekeeping and domestic economy of the institution. Experience showed that a trained nurse without experience in the latter departments often made a sad mess of the business management of the institution, and so fell into discredit. It required a large amount of tact to satisfy the medical staff, to manage the accounts, and to supervise the laundry and housekeeping. In the United States it was a common thing to find that the smaller the hospital the larger was the board of managers. One hospital contained 80 beds, and had 100 managers. A trained nurse in such circumstances discovered after a year or two that "she was totally unfitted for her proper duties, as she had exhausted her nerves, her brain, and her stomach, trying to train herself to be everything to every manager." It was imperative that the training schools at the larger hospitals should provide an extra course of three or six months, so that each graduate might have an opportunity of acting as assistant superintendent under conditions which would give her a general insight into the whole of the management, and so make her capable of exercising a wise control over all the departments of a hospital. This addition to a nurse's training would reduce the number of vacancies now caused by death. Mrs. Dennis stated that she had found a superintendent who was able to manage the small hospital of which she was president in the most satisfactory manner. She quite agreed that a short business training was essential in the case of those ladies who aspired to be superintendents. Miss Darche thought it was a great pity that some plan could not be devised which would ensure that the managers and directors of a hospital should in their turn be properly trained for the duties for which they were supposed to be responsible. This suggestion caused some laughter, but its point went home, and we commend it to the attention of all whom it may concern. There can be no doubt that if the managers and committee-men were subjected to a course of training they would not only be much more efficient, but much more attentive and zealous in the discharge of their duties. There are few hospitals which have not realised the importance of having a lady superintendent who is not only a trained nurse, but a woman of business capacity and tact.

### The Book World for Women and Nurses.

[We invite Correspondence, Criticism, Enquiries, and Notes on Books likely to interest Women and Nurses. Address, Editor, THE HOSPITAL (Nurses' Book World), 428, Strand, W.C.]

STUDIES BY A RECLUSE. By AUGUSTUS JESSOPP, D.D. (T. Fisher Unwin.)

There are some happy pens which have the gift of rendering whatever subject they approach pleasant, familiar, and easy to be understood. Like Charles Wesley, who could move a vast audience by his way of saying Mesopotamia, such guides, when the reader surrenders himself, make the choice of a subject indifferent. Whatever they choose to be interested in, they claim our attention for perforce. Dr. Jessopp touches in this volume on topics not usually approached in an hour of recreation. "The Land and its Owners in Past Times," "The Origin and Growth of English Towns," and the history of one or two of them, illustrated by the Monkish Chronicles and the surviving monuments, might prove in some hands very tough reading indeed. But two results may be safely prophesied for anyone who dips into these pages—first, that he will read it to the end with a refreshing sense of being brought into contact with living people which no modern society novel can lend, and secondly, that he will straightway look about for means to continue the stirring study of economic history to which he has been so ably introduced. The author has great confidence in the fascinations of history. Like Montaigne it is his "gibier en matière de lecture," and he would like everyone else to enjoy his game with him. In a modest passage in the preface he deprecates the title of historian; "for myself I prefer to be called a poacher in Clio's wide domains. They say you never can cure a rogue of poaching, it is born in him. I believe I shall go on poaching to the end; yes, as long as I can crawl. I have almost ceased to envy those favoured ones who have the run of the rich preserves and a dozen beaters at command, and the chance of sauntering through the covers when they please, their waistbands studded with cartridges. But let such keep their temper when they see me looking over the palings." Those who share the spoils with him will be inclined to regret that "the ringers and the singers, the sexton and the clerk, the relieving officer and the paupers, the guardians and the workhouse, the naughty old gossips and the saucy boys" have secured so much of the time which might have provided a more abundant feast. For it is just such "poachers" as himself, and they are few in number, who understand how to bring what is worth having into every common household. Dr. Jessopp seems to think almost anybody could do it, and advocates compulsory history teaching in the Board schools. But who does not know what a dreary thing the history lesson may be in unskilful hands? Better the children should grow up frankly ignorant than that they should spoil their chances of future enlightenment by the dismal early impression that they "don't like history." That impression, common to many persons who can boast of higher culture than that offered in the Board schools, can hardly be more satisfactorily corrected than by these light lectures and "studies," valuable chiefly as a stimulus to wider reading.

THE JULY REVIEWS.

THE conditions under which women's labour is too frequently carried on are forcibly exposed by Miss Marsh

Phillips in a review of the "Progress of Women's Trade Unions" in the Fortnightly. Insufficient and constantly decreasing pay and insanitary surroundings are the rule in countless centres of female occupation, and it is to remedy such abuses that a determined effort is being made to induce the women to combine in unions. A few examples are quoted. "In some factories the girls take their meals in rooms where the oranges are sorted and the cocoanuts smashed up. These are often very rotten, and the stench is such that the girls are sick, and have been sometimes even attacked by typhoid fever. Some of the work is quite unfit for women, for example, the carrying of tubs containing one cwt. of boiling syrup down a steep staircase all day long. Other girls do dangerous work at the mineral water trade, where accidents from bursting bottles occur at the rate of six a day. They work with caustic, which burns the skin off their faces, or ammonnia, which often makes them insensible, or they handle starch, which rots their boots and clothes and injures their lungs, and in only a few instances do the masters supply proper safeguards." The instances of starvation pay are even more pitiable, and the fact that in many trades men are losing their work on account of this cheap female labour adds another stringent reason for action of some kind in the best interests of the home. Whether the remedy can be looked for from the power of combination among these unhappy drudges is open to question. It would seem that any important movement for the better must come from without.

Cookery as a business is the theme of Miss Harrison in the Nineteenth Century, and amidst much that has been said a great many times before are some useful recommendations. The main point urged is the establishment of schools of cookery to which young girls could go on leaving school in preparation for good domestic service. It is suggested that the period of training should be three years, since it must ensure "not only instruction in the best methods, but repetition until the pupil can not only cook well, but to cook well has become a habit." This is no doubt very sensible and desirable, but what proportion of girls in our elementary schools, it must be asked, is in a position to undergo three years' training prior to beginning the business of life? Even were it possible to sacrifice three years of wage earning, it must be doubtful whether institution routine would afford anything like the practical training which subordination to a good cook in a private family can supply.

Many people will be surprised to learn from Mrs. Ritchie's Memories of Mrs. Kemble in Macmillan in what a curious groove of self-imposed rule much of her life was passed. "I can remember Mrs. Kemble sitting dressed in a black dress silently working all through the evening by her sister's fireside, and gravely stitching on and on while all the brilliant company came and went, and the music came and went. In those days Mrs. Kemble had certain dresses which she wore in rotation, whatever the occasion might be. If the black gown chanced to fall upon a gala day she wore it; if the pale silk gown fell upon a working day she wore it; and I can still hear an American girl exclaiming with dismay as the delicate folds of a white silk embroidered with flowers went sweeping over the anemones in the Pamphili gardens. "How do you suppose I could have lived my life," I once heard her say, "if I had not lived by rule, if I had not made laws for myself and kept to them?"

